# Advanced fetal cardiac monitoring in gestational diabetes mellitus: HbA1c remains the relevant predictor of perinatal outcome under optimal metabolic control

**DOI:** 10.1007/s00404-026-08427-x

**Published:** 2026-04-29

**Authors:** Leonie H. Kömmel, Helena Ortlam, Rebekka S. Loewe, Christian Schürer, Yvonne Heimann, Alexander Schmidt, Florentine Fiedler, Friederike Weschenfelder, Ekkehard Schleußner, Tanja Groten, Janine Zöllkau

**Affiliations:** 1 Department of Obstetrics, Jena University Hospital, 07747 Jena, Germany; 2 Department of Pediatrics, Jena University Hospital, 07747 Jena, Germany; 3 Department of Neurology, Biomagnetic Center, Jena University Hospital, 07747 Jena, Germany; 4 Center for Early Pregnancy and Reproductive Health, Jena University Hospital, 07747 Jena, Germany; 5 Department of Obstetrics, Medical Faculty and University Hospital Cologne, University of Cologne, Cologne, Germany

**Keywords:** Computerized cardiotocography, Fetal electrocardiography, Fetal heart time intervals, Heart rate variability, Maternal metabolic control

## Abstract

**Purpose:**

Perinatal complications can occur in gestational diabetes mellitus (GDM) despite adequate metabolic control and standard diagnostics. Metabolic alterations can cause structural and functional changes in the fetus, especially in the cardiovascular system, by affecting the autonomic nervous system and the cardiac conduction system. Advanced fetal cardiac monitoring may provide detailed insights into these processes and their impact on perinatal outcomes.

**Methods:**

In this exploratory, prospective, single-center cohort study, 172 women with singleton pregnancies between 33 + 0 and 40 + 0 weeks were recruited (56 GDM, 116 controls). Non-invasive fetal ECG (fECG) and computerized cardiotocography (cCTG) assessed the fetal heart rate variability (HRV) and heart time intervals (HTI). Adverse perinatal outcomes (APO) were defined as a composite of the clinically relevant endpoints of operative delivery or emergency cesarean for fetal distress, NICU admission, umbilical cord pH < 7.1, and/or 5-min APGAR < 7. Predictive potential was evaluated using univariate and multivariate regression models.

**Results:**

The median HbA1c in the GDM group was 5.32%, indicating overall good metabolic control. One hundred forty-five cCTGs and one hundred sixty-three fECGs provided data on HTI and fetal HRV parameters. HTI did not differ between GDM and controls. Although fetal HRV parameters differed, they did not add predictive value for APO. Only maternal metabolic status, as reflected by HbA1c, showed a measurable association with APO (OR 12.83, 95% CI 1.34–122.94).

**Conclusion:**

In well-controlled GDM pregnancies, HRV and HTI derived from fECG and cCTG do not enhance risk prediction for APO. Maternal HbA1c remains predictive for the perinatal risk, underscoring the importance of strict metabolic control.

## What does this study add to the clinical work

**Table:** 

This study shows that fetal cardiac monitoring does not enhance risk prediction in pregnancies with well-controlled gestational diabetes and highlights the importance of maternal metabolic management for favorable perinatal outcomes.	

## Introduction

Gestational diabetes (GDM) is the most common metabolic complication of pregnancy, with a rising prevalence of over 10% in pregnancies in Germany [1]. It results from an inadequate adaptation to pregnancy-related physiological changes, leading to relative insulin deficiency, maternal hyperglycaemia and fetal hyperinsulinemia. Therapeutic strategies, including lifestyle changes and pharmacological treatment, have been shown to improve maternal and perinatal outcomes by lowering blood glucose levels [2] and are widely recommended in clinical practice [3]. However, optimal glycaemic targets remain controversial, as current recommendations often exceed physiological glucose levels of non-diabetic pregnancies from mid-gestation onward [4]. International guidelines vary considerably and are mainly based on expert consensus rather than high-quality evidence [5–7].

Maternal hyperglycaemia exposes the fetus to excess glucose, impairing development and increasing the risk of perinatal complications and long-term metabolic and cardiovascular disorders [8, 9]. GDM also raises the risk of congenital heart disease and is linked to cardiac conditions such as cardiac hypertrophy [10, 11].

Analysis of the fetal electrocardiography (fECG) allows early detection of conduction abnormalities, such as those caused by cardiac hypertrophy or congenital heart diseases. The fECG morphology also allows the identification of fetal stress due to hypoxia or a metabolic acidosis. From the 32nd week of pregnancy, the quality of the fECG signal improves as gaps begin to form in the vernix caseosa, which has electrically insulating characteristics [12].

GDM is also reported to affect fetal autonomic development [13, 14]. The heart rate variability (HRV) is an established diagnostic and prognostic marker of the autonomic nervous system and allows detection of fetal autonomic nervous system dysfunctions [15]. The regulation of both the fetal heart rate (FHR) and HRV is overseen by the autonomic nervous system, which may be influenced by maternal glucose levels [16]. We demonstrated that there was an alteration in fetal autonomic control due to GDM and maternal blood glucose levels [14]. Furthermore, Fehlert et al. exhibited that alterations in FHR and HRV could be identified in women with GDM through fetal magnetocardiography (fMCG) monitoring. Consequently, monitoring HRV may serve as a method for detecting these changes in GDM patients [13, 14].

Our objective is to detect high-risk fetuses at an early stage and distinguish them from low-risk ones. This is crucial because, despite the availability of standardized diagnostics and prenatal screening, offspring of mothers with GDM still experience a higher rate of complications [9]. In this study, we challenged advanced assessment methods focusing on the fetal cardiac system to test the predictive potential of fetal HRV and heart time intervals (HTI) on neonatal outcome and to describe the dependence of cardiac electrophysiology and autonomic function on maternal metabolic control in GDM.

## Methods

From November 9, 2022, to October 12, 2023, this exploratory, prospective, monocentric, cohort study recruited pregnant women with GDM and healthy controls, each with singleton pregnancies between 33 + 0 and 40 + 0 weeks of gestation, at the Department of Obstetrics, Jena University Hospital, Germany. The targeted sample size, based on feasibility considerations, was 50 patients with GDM and 100 healthy controls, resulting in a 1:2 ratio. Exclusion criteria were pre-existing conditions such as type 1 or 2 diabetes, systemic lupus erythematosus, pre-eclampsia, symptomatic infections, cervical effective contractions, and fetal malformations; for controls, obesity was also excluded. GDM diagnosis followed IADPSG and WHO criteria [17] all participants were screened and treated according to German practice guidelines [5]. Obesity was defined and categorized according to the WHO criteria as body mass index (BMI) ≥ 30 kg/m^2^ [18].

The study was approved by the Ethics Committee of Friedrich Schiller University Jena. Participation was voluntary and written informed consent for both the participant and the newborns’ examination was obtained.

Clinical data reported were maternal age, BMI at pregnancy entrance, drug use and medication before and during the pregnancy, as well as previous illnesses. The perinatal outcome data included the birth weight, length, mode of delivery, APGAR score, umbilical artery and venous pH. An adverse perinatal outcome (APO) was defined as a composite of the clinically relevant endpoints of vaginal operative delivery or emergency cesarean due to fetal distress, NICU admission, umbilical artery pH < 7.1, and/or APGAR score at 5 min < 7.

Materno-fetal ultrasound examination and blood sampling were performed as part of routine care at our outpatient clinic in study inclusion. In addition, after delivery, umbilical cord blood samples were collected to analyze insulin and C-peptide as metabolic markers and NT-proBNP as a cardiac marker of the fetal compartment.

In terms of advanced fetal cardiac monitoring, the extended multimodal assessment consisted of a computerized cardiotocography (cCTG) for analysis of fetal HRV and an fECG for analysis of HTI and fetal HRV.

The fECG was recorded non-invasively via the mother’s abdominal wall using the AN24 device from Monica Healthcare Ltd. Nottingham, UK. The five electrodes were placed on the mother’s prepared abdomen in a standardized manner according to the manufacturer’s instructions [19]. In cases of a signal loss, the measurement was prolonged to ensure a total measurement length with a fetal signal of 30 min. The signal-averaged fECG complex was derived using MonicaDK, based on fetal signals isolated from the maternal ECG. The fECG records the fetal cardiac conduction and allows beat-to-beat analysis of the FHR as well as the determination of HTI.

The cCTG was recorded using Sonicaid software (Huntleigh Healthcare) at a 4 Hz sampling rate in a standardized manner. Signals were processed via advanced autocorrelation to derive FHR in beats per minute (bpm). Measurement continued until Oxford criteria were met, as indicated by the software [20], resulting in recordings of 15–45 min. A standardized HRV analysis was then performed using in-house Matlab-based software.

CTG data underwent semi-automatic artifact correction and fetal state classification by a trained medical student, followed by expert review by an obstetrician for accuracy. For artifact correction, the signal was segmented into 1-min intervals. Segments with > 20% of HR values outside 100–200 bpm or changes > 10 bpm between beats were excluded. The remaining signal sections (≥ 5 min) were rechecked using the same criteria. If outliers were detected, a stable FHR section was identified, defined as a segment where no criteria were violated over five consecutive heartbeats within 3 s. Segments with ≤ 10% artifacts were accepted. HRV parameters were calculated using a 5-min window shifted by 1 min and then weighted according to segment length [21].

Fetal state classification was based on basal HR and pattern, following Schneider et al. [22]. States included: 1F (deep sleep), 2F (active sleep), 3F (calm awake), and 4F (active awake) [23]. Due to limited clinical relevance, 3F was excluded [22]. Classification was first automated, then corrected by two trained individuals, with final decisions by a senior obstetrician.

A total of 102 HRV parameters were extracted per behavioral state and additionally in a state-independent analysis, resulting in an initial comprehensive dataset comprising 408 HRV parameters (102 parameters across four different fetal state categories). Due to the rare occurrence of state 4F, the selection process was focused on states 1F, 2F, and the state-independent data. A subsequent intended parameter reduction was performed prior to the final statistical analysis, based on physiological relevance and signal-analytical redundancy (correlation among HRV parameters), followed by group comparisons between pregnancies with GDM and controls, as well as between cases with and without APO. Furthermore, after reviewing and comparing the expression of HRV parameters and the distribution frequency of the fetal states and considering the clinical practicability of a potential future application, the subsequent statistical analyses were continued using only the state-independent parameter set. This approach resulted in a refined set of 15 representative parameters in the state-independent dataset that was subsequently used in the regression models, shown in Table 2 (appendix).

Statistical analyses were performed using SPSS 29.0 (IBM Corp. Released 2022. IBM SPSS Statistics for Macintosh, Version 29.0. Armonk, NY: IBM Corp). Due to the non-normal distribution of most of the data, median and interquartile range were used to describe the data. Non-parametric tests were used to compare continuous data between subgroups. During the evaluation of the methodology, correlation analyses as well as group comparisons were performed for rational parameter selection. Chi^2^ test and Mann–Whitney *U *Test were applied in this context where appropriate for the resulting data types. Final statistical analysis to assess the predictive potential of the selected HRV and HTI parameters in association with APO was performed using univariate and multivariate regression analyses. For each HRV, HTI, and outcome parameter, a separate regression model was constructed including the same predefined covariates (gestational age, pre-delivery BMI, and HbA1c at the study visit), with GDM diagnosis additionally included as a covariate in analyses of the overall cohort. This approach was used to adjust clinically relevant confounders, predescribed in the literature, known to influence perinatal outcome [9, 24, 25]. Due to the exploratory study design, adjustments to the alpha level for multiple comparisons were not performed. Statistical significance was determined at *p* < *0.05* and statistical trend at *p* < *0.1*.

## Results

**Fig. 1:**
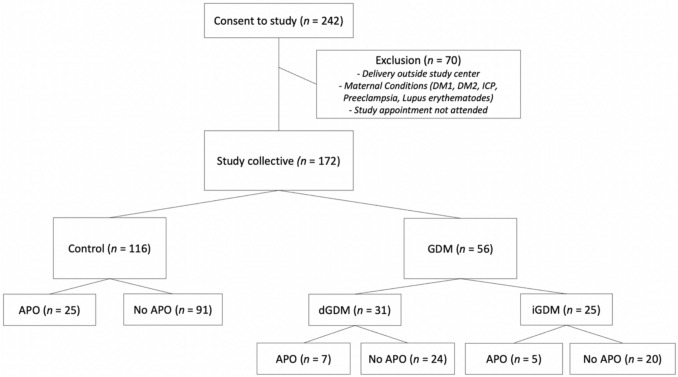
Cohort composition of 242 women who consented, 172 were included: 116 in the control cohort and 56 in the GDM cohort (31 diet-controlled [dGDM], 25 insulin-dependent [iGDM]). Adverse perinatal outcomes (APO; combined endpoint of operative delivery/emergency cesarean due to fetal distress, NICU admission, umbilical cord pH < 7.1, APGAR Score at 5 min < 7) occurred in 25 controls, 7 dGDM, and 5 iGDM cases

Taking into account the inclusion and exclusion criteria presented, 172 women and their children were part of the analysis. The composition of the study cohort regarding the diagnoses of GDM and APO is shown in Fig. 1.

GDM was diagnosed in 56 women (32.6%). Of these, 21 women had a BMI ≥ 30 (37.5%). The control cohort consisted of 116 women. Patient and birth characteristics in comparison between GDM and controls are summarized in Table 1.

**Table 1:** Summary of the accompanying data of the GDM (gestational diabetes mellitus) cohort and comparison to the controls

	Available data (control/GDM)	Control	GDM
Number of participants		116	56
Patient characteristics			
Age	(116/56)	32 (29–35)	33 (30–35)
Gravida	(116/56)	2 (1–2)	2 (1–3)
Para	(116/56)	1 (0–1)	0.5 (0–1)
BMI (kg/m^2^) preconceptual	(116/56)	22.35 (20.3–24.9)	27.35 (23.75–31.7)
High risk pregnancy*	(116/56)	35 (30.2%)	22 (39.3%)
Weight gain in pregnancy (kg)	(116/56)	15 (12–18)	10.5 (7.5–15)
Birth characteristics			
Gestational age at delivery	(116/56)	39 + 4 (38 + 2–40 + 0)	39 + 6 (39 + 0–40 + 4)
Labor induction	(116/56)	27 (23.3%)	21 (37.5%)
Preterm birth (< 37 weeks)	(116/56)	1 (0.9%)	3 (5.4%)
Delivery mode	(116/56)		
Spontaneous		70 (60.3%)	39 (69.6%)
Vaginal operative		8 (6.9%)	1 (1.8%)
Primary cesarean section		13 (11.2%)	7 (12.5%)
Emergency cesarean section		25 (21.6%)	9 (16.1%)
Surgical delivery due to fetal stress	(114/56)	16 (14%)	5 (8.9%)
CTG in labor suspect**	(114/56)	6 (5.3%)	4 (7.1%)
CTG in labor pathological**	(114/56)	31 (27.2%)	13 (23.2%)
MBU necessary	(116/56)	8 (6.9%)	6 (10.7%)
Placental weight	(74/49)	464.5 (400–520)	485 (440–554)
Birth length (cm)	(116/56)	51 (50–52)	51 (49–54)
Birthweight (g)	(116/56)	3360 (3085–3685)	3525 (3122.5–3750)
APGAR 5 min < 7	(116/56)	3 (2.6%)	2 (3.6%)
pH umbilical cord	(115/55)	7.22 (7.18–7.28)	7.23 (7.17–7.3)
Base excess umbilical cord (mmol/l)	(116/56)	–5.35 (–7.7– (–2.15))	–3.95 (–7.05– (–1.65))
Respiratory distress syndrome	(116/56)	8 (6.9%)	5 (8.9%)
NICU admission	(116/56)	11 (9.5%)	5 (8.9%)
Adverse perinatal outcome (APO)	(116/56)	25 (21.6%)	12 (21.4%)
Study measurement			
Gestational age study inclusion	(116/56)	36 + 1 (34 + 5–36 + 4)	35 + 3 (34 + 0–36 + 2)
Gestational age fECG	(108/55)	36 + 2 (35 + 1–37 + 2)	36 + 0 (34 + 2–37 + 0)
Gestational age cCTG	(116/56)	36 + 2 (35 + 2–37 + 2)	35 + 6 (34 + 1–37 + 0)
Estimated fetal weight percentile in prenatal ultrasound at visit	(101/56)	40 (25–62)	62.5 (40.5–82)
Abdominal circumference percentile in prenatal ultrasound at visit	(101/56)	38 (25–63)	57 (37–77.5)
Birth weight (*g*)	(116/56)	3360 (3085–3685)	3525 (3122.5–3750)

Data are n (%) or median (interquartile range); percentages are based on available data for each variable; Adverse perinatal outcome is a combined endpoint (operative delivery /emergency cesarean due to fetal distress, NICU admission, umbilical cord pH < 7.1 and/or APGAR Score at 5 minutes < 7); *fECG*, fetal electrocardiogram; *cCTG*, computerized cardiotocography; *nECG*, neonatal ECG * According to national maternity record criteria; ** according to FIGO criteria

Analyses of patient subgroup characteristics indicated no further differences regarding ethnicity, smoking, medication and drug use, incidence of abortion and intrauterine fetal demise, and pre-existing medical conditions. However, the pre-pregnancy BMI was notably lower in the control cohort, a phenomenon attributable to the study design, in which maternal obesity was excluded a priori from the control group. In prenatal ultrasound assessments, abdominal circumference (*p* = 0.01) and estimated fetal weight (*p* < 0.001) differed significantly between the GDM cohort and the control group, whereas no significant difference was observed in birth weight. The significant differences in gestational age at study enrollment and during measurements do not hold clinical relevance in terms of absolute gestational age.

**Fig. 2:**
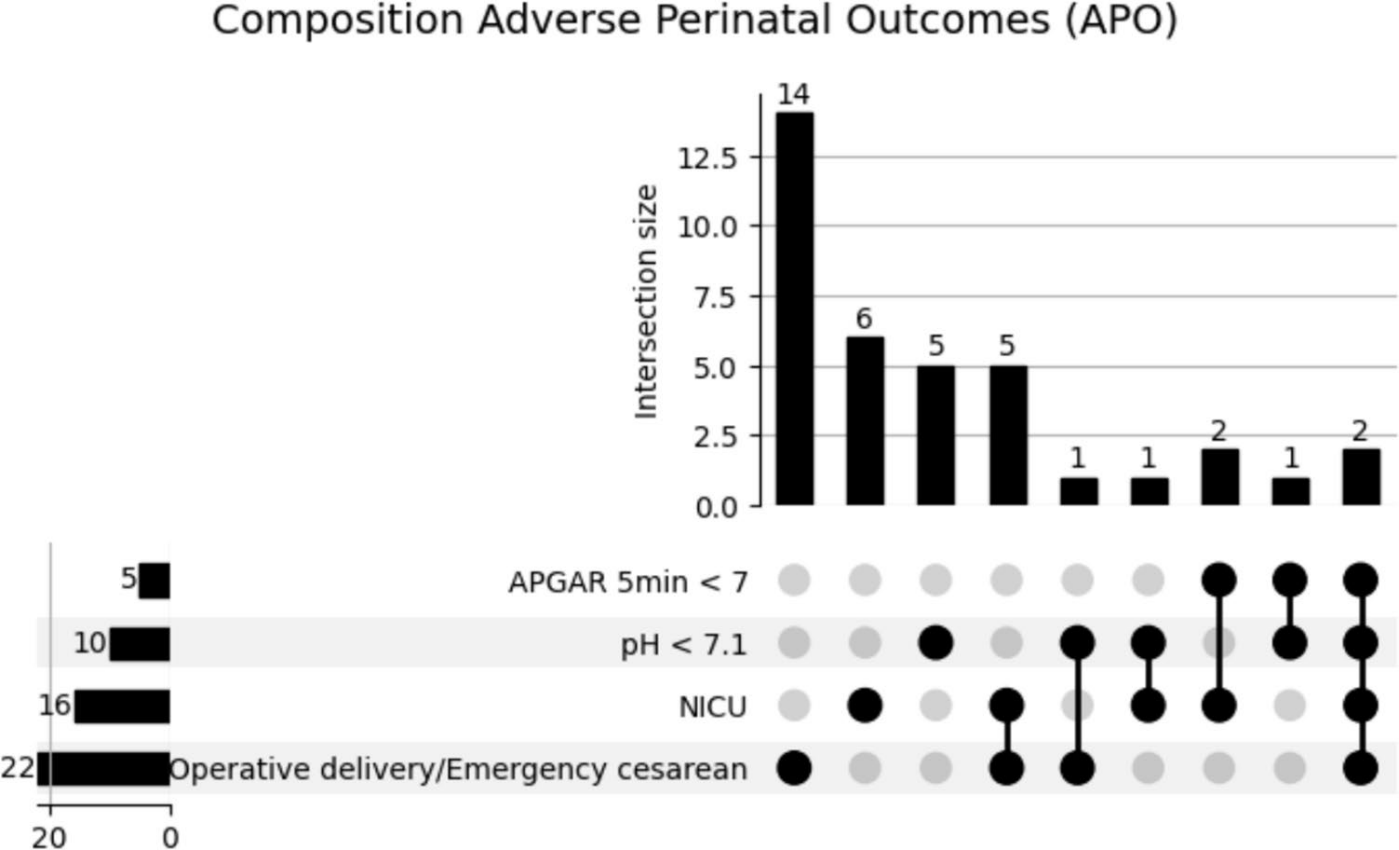
Composition of adverse perinatal outcome in the total cohort: adverse perinatal outcomes (APO; combined endpoint of operative delivery/emergency cesarean due to fetal distress, NICU admission, umbilical cord pH < 7.1, and/or APGAR Score at 5 min < 7). A total of 25 cases in the control group and 12 in the GDM group experienced one or more of these events, in various combinations

APO occurred in 37 (21.5%) of all analyzed pregnancies as seen in Fig. 2, from which 25 (21.6%) occurred in the control cohort and 12 (21.4%) in the GDM cohort, and there was no significant difference in the occurrence of APO across the subgroups. Notably, both cases in which all components of the composite endpoint occurred were observed in the GDM cohort. One neonatal death occurred on the 8th day of life, which was associated with a preclinical uterine rupture in the 39th week of gestation and within the control cohort.

The cCTG was performed in all study participants. During cCTG processing, 27 cCTGs were excluded due to artifact correction, 15 in the control and 12 in the GDM cohort. Of the 145 cCTGs subjected to artifact correction, 14 showed phases with state 1F pattern, 126 showed phases with state 2F pattern, and 5 showed phases with state 4F pattern. Due to the rare occurrence of state 4F, the analysis was focused on 1F, 2F and state-independent data. The fECG was performed in 163 participants, 55 (98.2%) in GDM and 108 (93.1%) in the control cohort. Blood samples, including HbA1c measurements, were obtained from 113 (97.4%) participants in the control cohort and 56 in the GDM cohort (100%).

**Fig. 3:**
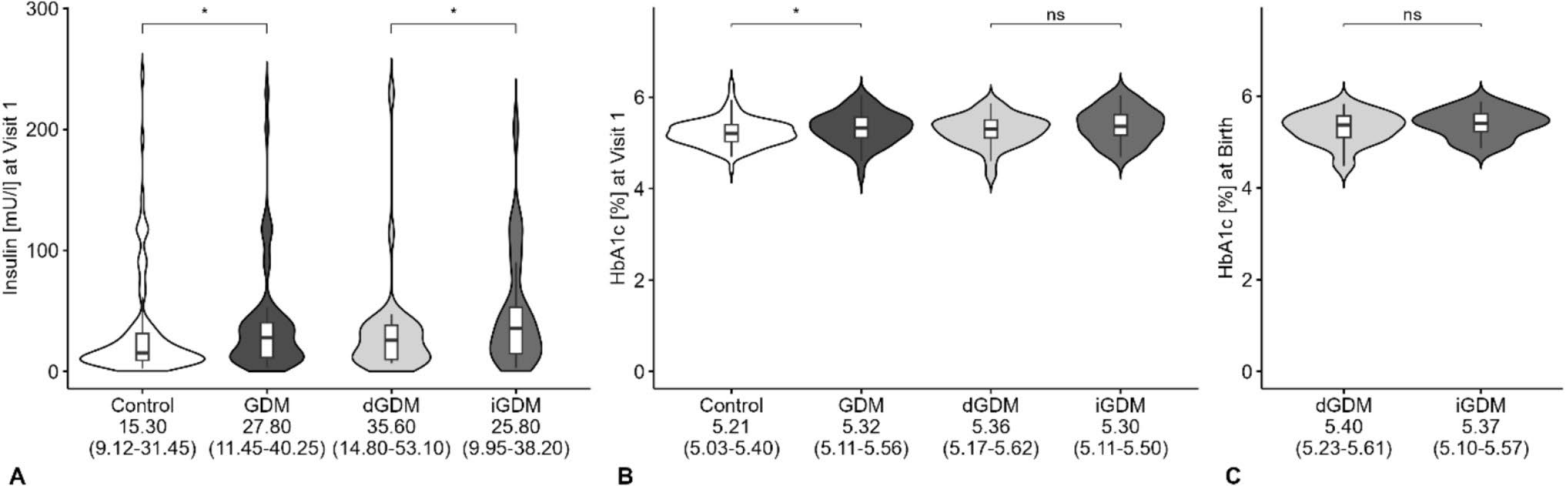
Maternal metabolic control in the study cohort: *GDM*, gestational diabetes mellitus; *iGDM*, insulin-managed GDM; *dGDM*, dietary-managed GDM; **A** insulin at Visit 1 differed between control and GDM (*p* = 0.023) and between dGDM and iGDM (*p* = 0.046); **B** HbA1c at Visit 1 differed between control and GDM (*p* = 0.032), but not between dGDM and iGDM (n.s.); **C** HbA1c at birth showed no difference between dGDM and iGDM (n.s.); * significant result < 0.05, n.s., non-significant result (statistical comparison by Mann–Whitney U Test). Displayed below are medians (interquartile range)

Regarding the metabolic control in the GDM cohort, the median HbA1c was 5.32% as shown in Fig. 3, well below the critical level of 5.7% [26]. Although the GDM cohort showed a significantly higher mean HbA1c (*p* = 0.032), no significant difference between the GDM and control groups was observed when applying an HbA1c cutoff of ≥ 5.7% (*p* = 0.233). In addition, fetal metabolic markers measured in umbilical cord blood revealed significantly higher insulin (9.2 vs 6.7mU/l; *p* = 0.007) and C-peptide levels (1.5 vs 1.2; *p* = 0.006) in the GDM cohort compared with the control group. Furthermore, NT-proBNP levels in umbilical cord blood, analyzed as a marker of fetal cardiac status, also differed significantly between the GDM and control cohorts (748.5 vs 652; *p* = 0.007).

**Fig. 4:**
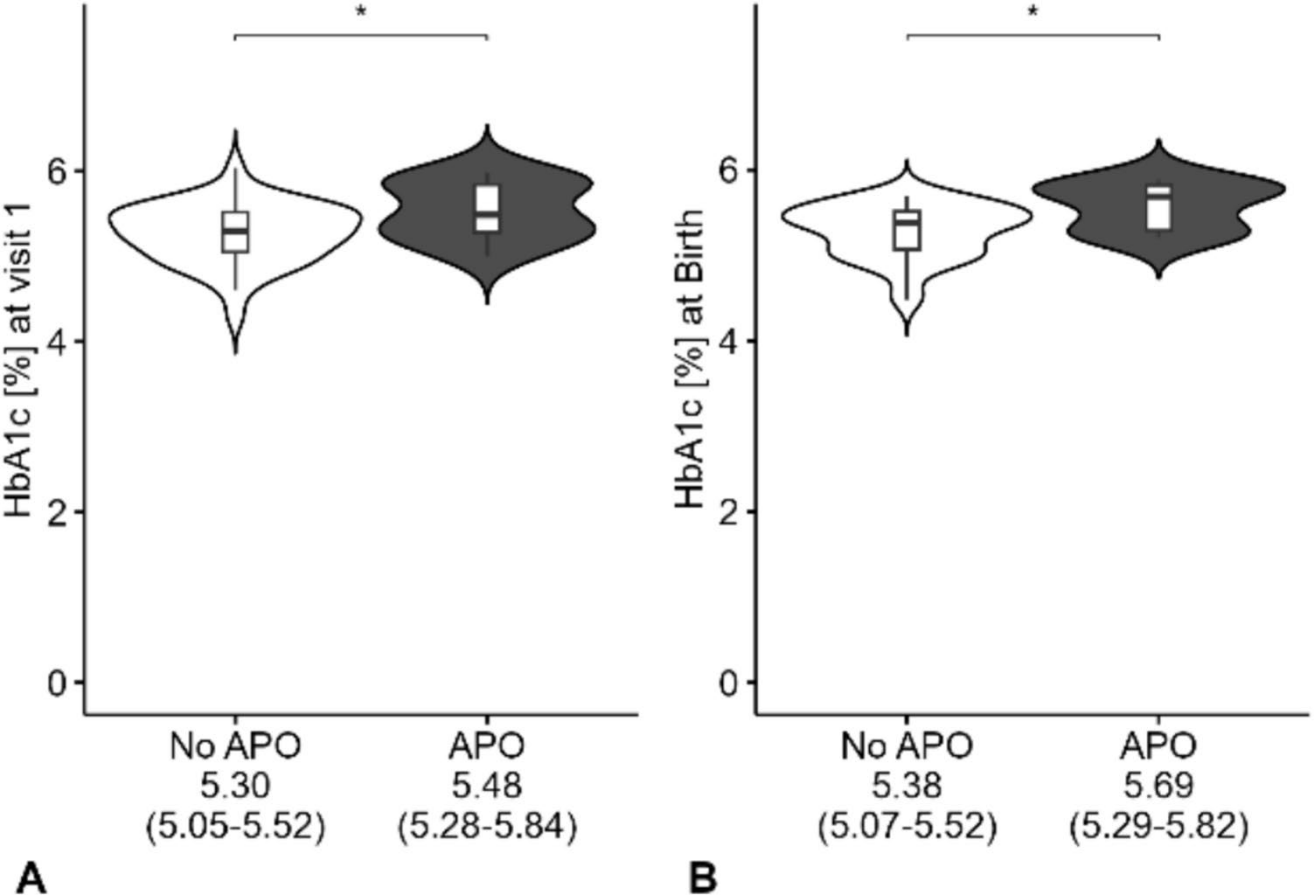
Maternal metabolic state in dependence on the perinatal outcome in GDM (gestational diabetes mellitus) cohort: *APO*, adverse perinatal outcomes (combined endpoint of operative delivery/emergency cesarean due to fetal distress, NICU admission, umbilical cord pH < 7.1, APGAR Score at 5 min < 7): **A** HbA1c at Visit 1 differed between No APO and APO (*p* = 0.045); **B** HbA1c at birth differed between No APO and APO (*p* = 0.0017); * significant result < 0.05 (statistical comparison by Mann–Whitney *U* Test). Displayed below are medians (interquartile range)

In comparative analyses, the expectant mothers with GDM whose offspring had APO demonstrated statistically significant elevations in HbA1c levels compared to those with favorable outcomes, both during the study visit (*p* = 0.045) and at the time of delivery (*p* < 0.01) as shown in Fig. 4. No significant differences in umbilical cord blood parameters were found between neonates with and without APO.

Within the GDM cohort, there were no significant differences in HRV parameters related to metabolic control (cutoff HbA1c ≥ 5,7%). In the fECG, the QT-time was significantly shorter (median 170.5 vs. 198.5, *p* = 0.021) in the GDM subcohort with poor metabolic control.

**Fig. 5:**
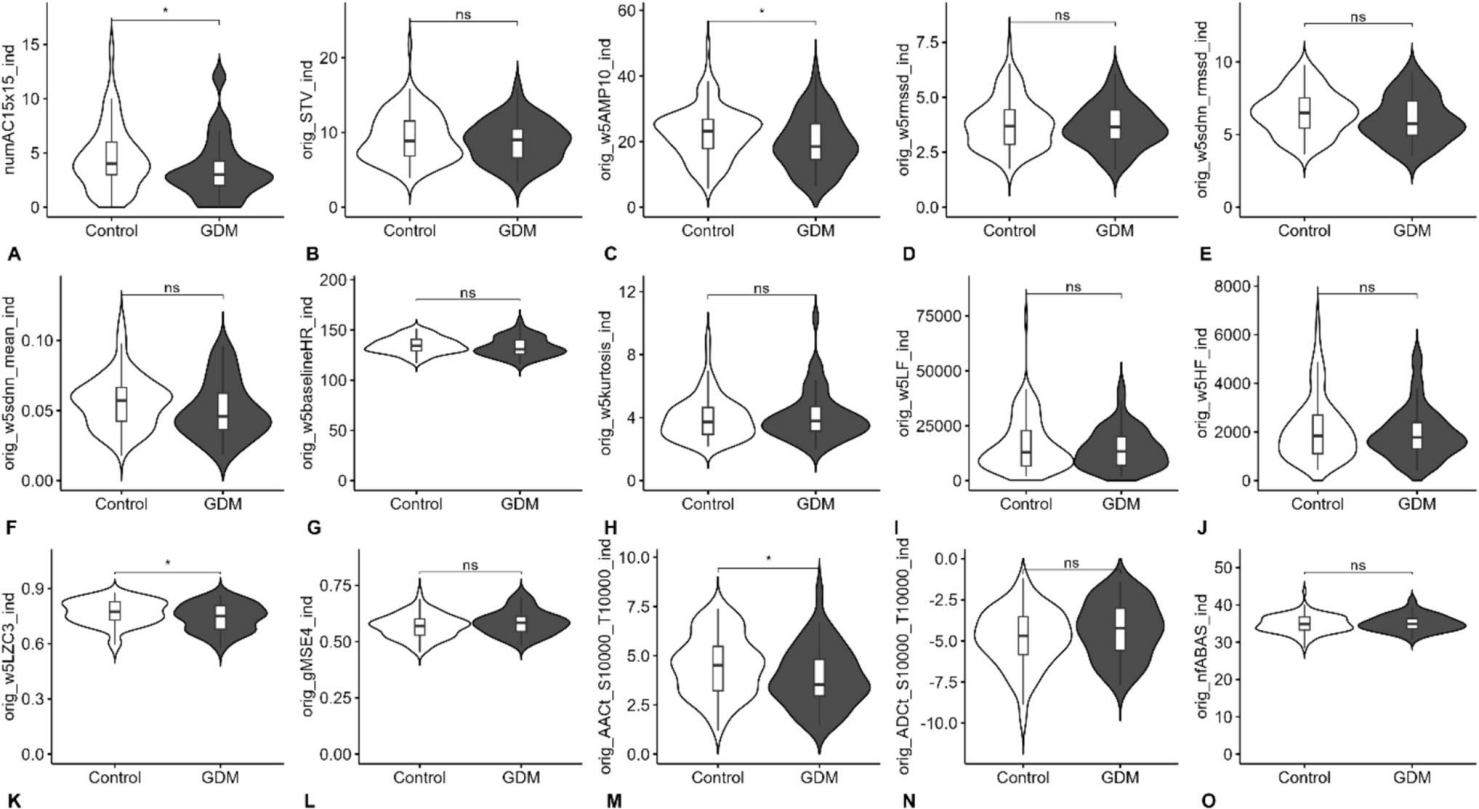
cCTG (computerized cardiotocography)-derived fHRV (fetal heart rate variability) parameters in the control and GDM cohort. Data are median (interquartile range), unless otherwise specified; *GDM* gestational diabetes mellitus, *cCTG*: computerized cardiotocography, **A** accelerations (*p* = 0.022); **B** short-term variability; **C** amplitude range (*p* = 0.037); **D** RMSSD; **E** ratio of sdnn to rmssd; **F** sdnn; **G** baseline heart rate; **H** kurtosis; **I** low-frequency band power; **J** high-frequency band power; **K** complexity parameter (*p* = 0.028); **L** complexity parameter; **M** acceleration capacity (*p* = 0.031); **N** deceleration capacity; **O** functional fetal autonomic brain age score * significant result < 0.05, *n.s.* non-significant result (statistical comparison by Mann–Whitney U Test)

Comparing the differences in cCTG and fECG parameters between the GDM and control cohorts, only a few trends and significant differences were observed as shown in Fig. 5. The GDM group exhibited a reduced acceleration capacity (*p* = 0.031). Differences in complexity parameters (*p* = 0.028) were also observed, with some indicating greater complexity in the GDM cohort and others indicating greater complexity in the control cohort.

**Fig. 6:**
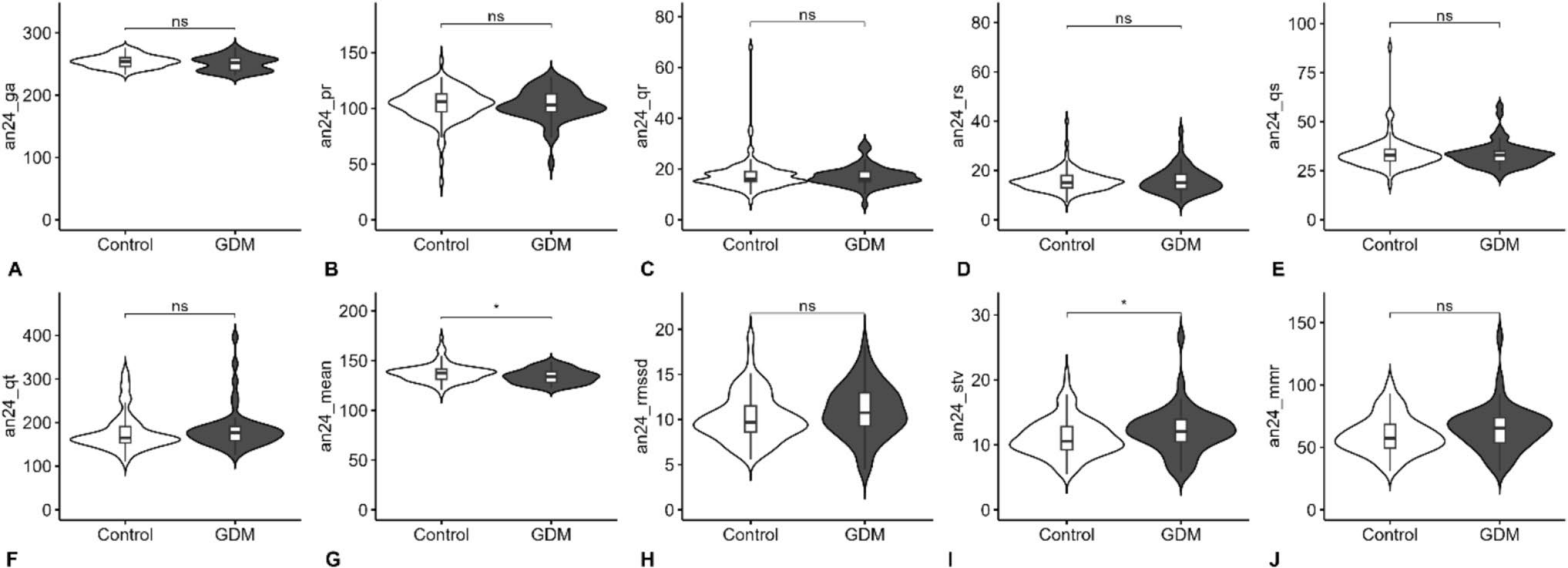
fECG (fetal electrocardiogram) parameters in the control and GDM cohort. Data are median (interquartile range), unless otherwise specified; *GDM*, gestational diabetes mellitus; *fECG*, fetal electrocardiogram; **A** gestational age; **B** PR-time; **C** QR-time; **D** RS-time; **E** QRS-time; **F** QT-time; **G** mean heart rate (*p* = 0.017); **H** RMSSD; **I** short-term variability (*p* = 0.046); **J** mean minute range; * significant result < 0.05, n.s., non-significant (statistical comparison by Mann–Whitney U Test)

In the fECG, significant differences were observed only in HRV parameters between the control and GDM cohorts, which are shown in Fig. 6, while no significant differences were found in HTI or amplitudes. The GDM group exhibited a higher short-term variability (STV) (*p* = 0.046), a lower mean HR (*p* = 0.017) and basal FHR (*p* = 0.028).

**Fig. 7:**
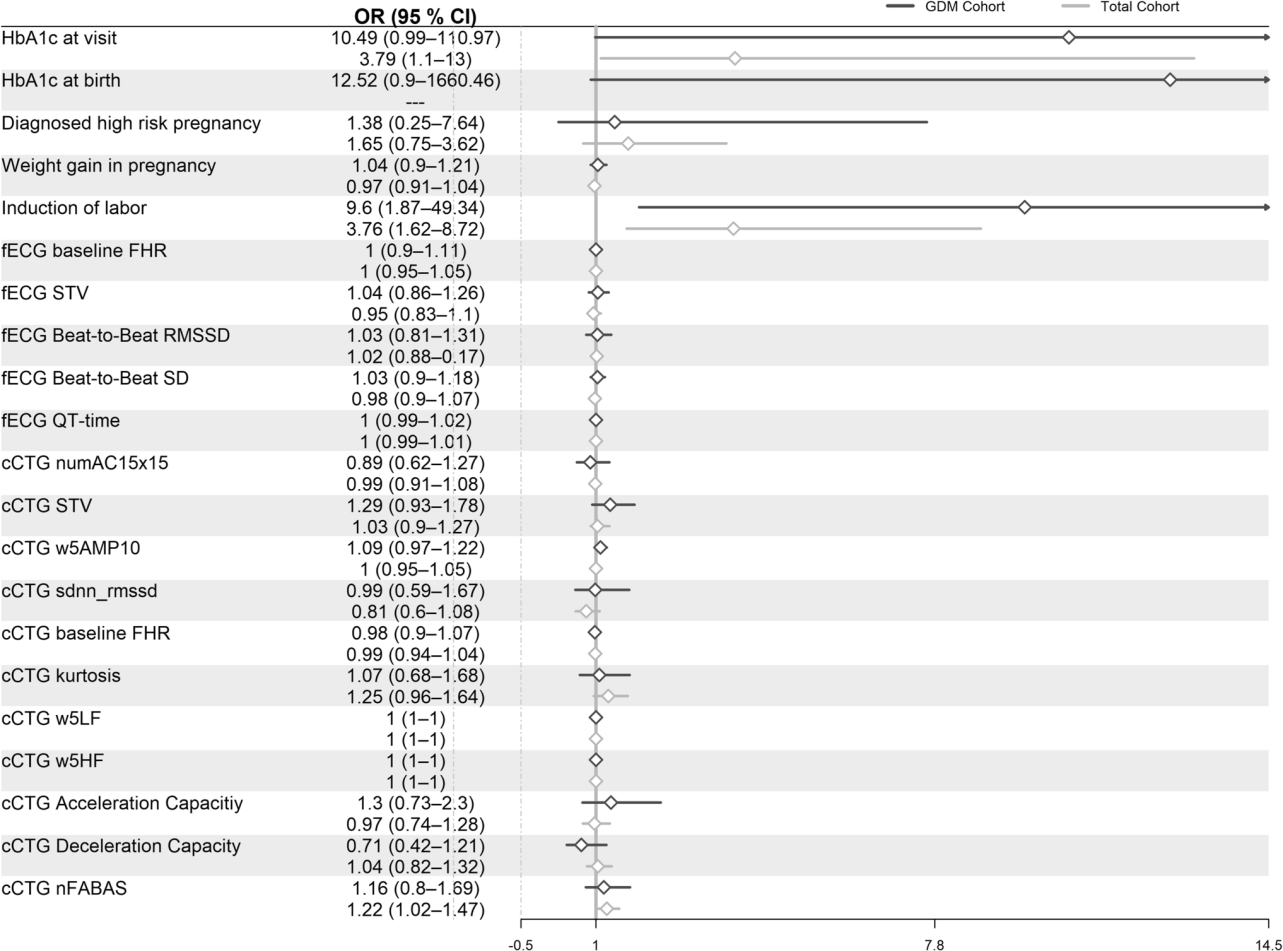
Predictive capacity of the selected fECG (fetal electrocardiogram) and cCTG (computerized cardiotocography) parameters individually adjusted for gestational age at delivery, maternal BMI, and HbA1c at study visit and additionally for GDM in the total cohort, *each row represents one generalized linear model* *GDM*, gestational diabetes mellitus, *fECG*, fetal electrocardiogram, *cCTG*, computerized cardiotocography, *FHR*, fetal heart rate, *STV*, short-term variation, *numAC15 × 15*, acceleration, *w5AMP10*, amplitude range, *w5LF*, low-frequency band power, *w5HF*, high-frequency band power, *nFABAS*, functional fetal autonomic brain age score

In univariable regression analysis within the GDM cohort, the HbA1c at Visit 1 (*p* = 0.027) and at the point of birth (*p* = 0.024) had a significant influence on fetal outcome. Within the overall cohort, univariable regression analysis indicated an increased risk associated with HbA1c at Visit 1 (*p* = 0.032) and also the HRV parameters nfABAS (functional fetal autonomic brain age score) (*p* = 0.033) and complexity (gMSE4) (*p* = 0.022). Subsequently, we evaluated the predictive capacity within the overall cohort by including HbA1c and GDM diagnosis as covariates to assess whether these variables could contribute to the prediction of APO in the whole cohort. Multivariable regression analyses revealed no significant differences in HTI parameters and in only one HRV parameter, the nFABAS (*p* = 0.034), as shown in Fig. 7. However, HbA1c levels at the study visit showed a significant association with fetal outcome only in the overall cohort (*p* = 0.034), but not within the GDM subgroup, as shown in Fig. 7. Induction of labor was also shown to increase the likelihood of APO in univariable and multivariable regression analyses in the GDM (*p* = 0.005; *p* = 0.007) and the overall cohort (*p* < 0.001; *p* = 0.002).

## Discussion

### Main findings

In this prospective, monocentric, cohort study, 172 pregnant women (56 with GDM, 116 controls) underwent advanced fetal cardiac monitoring using cCTG and fECG. Maternal metabolic control was monitored throughout the study. HRV and fECG-derived parameters were analyzed for group differences and their predictive value for APO. Additionally, the impact of maternal metabolic control on APO occurrence and the examined parameters was evaluated. Prediction of APO was not possible in the overall population or in the analyzed population of well-controlled GDM pregnant women with the methods used. In addition to the near-normal glycaemic control observed in the GDM cohort as a possible explanation, the incidence of APO was similar between the GDM and control cohorts. Both aspects must be considered when interpreting the subsequent regression results, as fetal cardiac alterations that may be pathophysiologically related to elevated maternal blood glucose levels would be expected to occur less frequently in such a cohort.

Univariable regression analysis indicated an increased risk associated with higher nfABAS and gMSE4 in the study cohort. Caution is warranted in interpreting these results, as the majority of cCTG-based HRV parameters did not show significant differences. This finding is also physiologically unexpected, as greater autonomic nervous system maturity is generally associated with favorable physiological conditions [27, 28].

### Strengths and limitations

Given the large number of HRV parameters initially analyzed, the issue of multiple testing represents an important methodological consideration. As our study followed an exploratory design aimed at identifying potentially predictive parameters rather than confirming predefined hypotheses, no formal correction for multiple testing was applied. We acknowledge that the evaluation of a large number of parameters increases the risk of type I errors due to multiple comparisons. To mitigate this risk within the statistical analysis strategy, we implemented a structured and rationale-based parameter selection process prior to the final analyses, reducing the dataset to a limited number of physiologically and signal-analytically representative HRV parameters. This step was intended to limit redundancy among highly correlated HRV measures and to reduce the number of statistical tests performed. At the same time, applying a strict alpha correction in this exploratory context could have substantially increased the risk of type II errors, potentially obscuring meaningful associations that may warrant further investigation. Therefore, the results should be interpreted with appropriate caution, and the identified associations should be considered hypothesis-generating and be validated in future, adequately powered confirmatory studies.

A limitation of our study is the exclusive use of HbA1c as a uniform glycaemic control marker, since other parameters (e.g., TIR, MBG) were inconsistently collected and thus not comparable. In the interpretation of our findings, it must therefore be considered that HbA1c can only serve as an indirect marker of fetal metabolic exposure. Notably, seven individuals (6.2% of those with available HbA1c data [*n* = 113]) in the control group showed HbA1c ≥ 5.7%, suggesting potential gaps in current screening and possible undetected cases of impaired glucose metabolism [29, 30]. Furthermore, 21 individuals (37.5%) in the GDM group fulfilled WHO obesity criteria with BMI ≥ 30 kg/m^2^ while obesity was excluded in the healthy controls. Both factors lead to a limited degree of selection bias, which must be considered when interpreting the exploratory findings. On the other hand, obesity is associated with metabolic alterations independent of GDM, including insulin resistance and low-grade inflammation [31], which may affect fetal development and autonomic regulation. Therefore, excluding obesity in the control group enabled the definition of a metabolically healthy reference population and minimized confounding. Moreover, the sample size, which was determined based on feasibility considerations within the framework of an exploratory pilot study, as well as the resulting limited number of APO cases available for statistical analyses, represents a limitation and should be taken into account when interpreting our results. The composite APO used in this study comprises clinically heterogeneous endpoints; however, it was intentionally defined based on clinical experience with pregnancies complicated by GDM to capture a spectrum of adverse events that are frequently observed despite guideline-based management but for which reliable prenatal predictors are currently lacking. As this study was conducted in a single-center cohort at a specialized perinatal center, the generalizability of the findings to other populations or clinical settings may be limited and should therefore be interpreted with caution.

This prospective study’s strength lies in advanced multimodal fetal monitoring using non-invasive fECG and cCTG, enabling comprehensive assessment of fetal cardiac activity and autonomic function. Standardized data collection in a homogeneous, well-controlled GDM cohort enhances internal validity, while combining two HRV measurement methods allows robust evaluation of signal quality and reproducibility.

### Interpretation

In contrast to previous studies reporting altered fetal autonomic or cardiac function in GDM pregnancies [13, 14, 32], we observed no significant differences in fetal parameters between the GDM and control groups in our well-managed cohort. Consistent with this, the incidence of APO was comparable between the GDM and control cohorts, likely reflecting the good metabolic control in the GDM cohort and explaining the absence of differences in fetal HRV or fECG-derived parameters. Similarly, no significant differences were observed in fetal growth outcomes, including birth weight and the proportion of macrosomic fetuses based on ultrasound-derived estimated fetal weight percentiles. Although umbilical cord blood analysis showed significant differences in fetal metabolic markers between groups, these were not associated with APO and therefore did not indicate clinically relevant differences in neonatal outcome. Findings similar to ours were reported in the study recently published by Chivers et al., which found no group differences between the GDM and control cohorts in fetal HRV, FHR, or HTIs, also under conditions of excellent glycemic control in the GDM cohort [33]. This consistency across studies suggests that strict maternal metabolic control may mitigate fetal cardiac alterations otherwise attributed to GDM. In cohorts with more relevant metabolic impairments, differences in the fECG and cCTG parameters might be observed, since we were able to show that poor maternal metabolic control leads to a poor fetal outcome. A further common factor was the timing of the initial measurements, which occurred at 36 weeks of gestation in our study and at 34 weeks in the study by Chivers et al. Consequently, a considerable time interval existed between the diagnosis of GDM in the 24th week of pregnancy, the initiation of treatment, and the measurement of fetal parameters. This period may have been sufficient to improve the underlying metabolic dysfunction associated with GDM, potentially mitigating its impact on FHR and HRV [33].

Clinically, our analysis highlights HbA1c as a relevant predictor of neonatal outcomes, both in GDM and the overall cohort. These findings suggest that strict glycemic control in pregnancies affected by GDM remains a crucial factor in ensuring optimal neonatal health. To date, there is a lack of high-quality studies directly comparing different glycemic targets in the treatment of GDM [34]. Several proposed postprandial glycemic targets [2, 35] exceed the postprandial glucose levels observed in non-diabetic individuals [4]. Notably, one study reported a reduction in serious neonatal outcomes including perinatal mortality, birth trauma, and shoulder dystocia as well as shorter neonatal intensive care unit stays among infants of mothers treated with stricter glycemic targets [36]. These findings highlight the potential value of reassessing current target thresholds in GDM treatment.

Consistently, GDM did not emerge as an independent risk factor in our multivariable analyses, reinforcing that adequate metabolic control effectively minimizes perinatal risk [2]. This may also be related to the fact that the women who took part in the study attended additional appointments and examinations and are therefore more likely to be regarded as compliant. Furthermore, there was no difference in the incidence of APO between the GDM and control cohorts, indicating a well-managed and closely monitored study population.

## Conclusion

In pregnancies complicated by GDM with well-controlled metabolism, advanced fetal cardiac monitoring using cCTG and fECG did not provide additional predictive value for APO. The decisive determinant of neonatal health was maternal metabolic control, with HbA1c emerging as predictive for the perinatal outcome. Maintaining strict glycemic regulation, therefore, remains essential to optimize perinatal outcomes in GDM. These findings also underline the importance of critically reassessing current glycemic targets, as stricter thresholds may provide further clinical benefit.

Future studies should, therefore, focus on earlier gestational ages, particularly in women with insufficient glycemic control or newly diagnosed GDM, include a more detailed glycemic metabolic characterization of mothers and neonates, and potentially combine autonomic monitoring with structural fetal metabolic phenotyping as an important next step. This approach would allow detection of potential effects of high-risk individuals, inadequate therapeutic adherence or newly diagnosed GDM on fetal autonomic regulation and would assess whether timing and intensity of metabolic stress influence outcomes differently. Larger, confirmatory studies are also needed to evaluate whether refined monitoring approaches can provide predictive value in higher-risk populations and whether alternative metabolic markers beyond HbA1c may improve risk stratification.

## Data Availability

No datasets were generated or analyzed during the current study.

## Appendix

See Table 2.

**Table 2:** Refined cCTG (computerized cardiotocography) parameter set

Parameter	Domaine	Category	Meaning
cCTG numAC15 × 15	Morphological	Pattern	Accelerations of different bpm-magnitude x sec-duration
cCTG STV	Linear	lAMP	Short-term variability according to CTG
cCTG w5AMP10	Linear	lAMP	Amplitude range: 10–95 interquartile distance of detrended NN interval series or CTG samples
cCTG RMSSD	Linear	sAMP	Root mean square of successive NN interval or CTG sample differences
cCTG SDNN	Linear	lAMP	Standard deviation of NN intervals or CTG samples
cCTG sdnn_rmssd	Linear	SVB	Ratio of sdnn to rmssd
cCTG baseline FHR	Linear	lAMP	MeanHR based on the estimated baseline
cCTG kurtosis	Linear	Pattern	Kurtosis of NN intervals or CTG samples
cCTG w5LF	Frequency	lAMP	Low-frequency band power
cCTG w5HF	Frequency	sAMP	High-frequency (0.4–1.7 Hz) band power
cCTG LZC3	Nonlinear	sCOMP	Lempel ziv complexity of ternary transformed NN intervals or CTG samples
cCTG gMSE4	Nonlinear	sCOMP/lCOMP	Multiscale entropy at specific coarse graining levels, calculated using generalized mutual information or sample entropy
cCTG acceleration capacity	Nonlinear	sAMP/lAMP	Averaged acceleration capacity of different second steps obtained by phase rectified signal analysis for NN-interval-based data
cCTG deceleration capacity	Nonlinear	sAMP/lAMP	Averaged deceleration capacity of different second steps obtained by phase rectified signal analysis for NN-interval-based data
cCTG nFABAS	MM	Score	Functional fetal autonomic brain age score based on normalized/log transformed HRV-features
